# Plasma Carrier Proteins as Supramolecular Nanovectors for Cancer Therapy: The Trojan Horse Approach

**DOI:** 10.3390/cancers18193106

**Published:** 2026-09-24

**Authors:** Alessia Marconi, Andrea Carboni, Matteo Di Giosia, Matteo Calvaresi

**Affiliations:** 1 Preclinical & Translational Research in Oncology Lab (PRO), IRCCS Azienda Ospedaliero—Universitaria di Bologna, 40138 Bologna, Italy; alessia.marconi@aosp.bo.it (A.M.); andrea.carboni9@unibo.it (A.C.); matteo.digiosia2@unibo.it (M.D.G.); 2 Dipartimento di Chimica “Giacomo Ciamician”, Alma Mater Studiorum—Università di Bologna, Via Piero Gobetti, 85, 40129 Bologna, Italy

**Keywords:** chemotherapeutics, plasma proteins, “Trojan Horse”, drug delivery systems, nanocarriers, tumor targeting strategies, nanomedicine, personalized medicine

## Abstract

Conventional chemotherapeutics often suffer from poor aqueous solubility, short circulation half-lives, lack of tumor selectivity, and present dose-limiting systemic toxicity. This perspective examines the Trojan Horse Approach (THA), in which plasma carrier proteins (PCPs) are exploited as discrete, non-covalent supramolecular hosts for chemotherapeutic agents, and discusses its potential to overcome the limitations of both free drugs and conventional protein nanoparticles. Two principal strategies for assembling ex vivo drug–protein complexes are discussed: (i) molecular recognition, in which drugs occupy native ligand-binding pockets, and (ii) molecular anchoring, in which drugs are conjugated to high-affinity anchor ligands. The perspective critically assesses the current state of the art, distinguishes different levels of experimental validation, and discusses the major limitations and barriers to clinical translation. Structural, biochemical, and pharmacological evidence is examined for human serum albumin, transferrin, transthyretin, alpha-1-acid glycoprotein, alpha-fetoprotein, lactoferrin, and other plasma proteins. When appropriately designed and validated, protein-based complexes may preserve the native protein structure while conferring improved drug solubility, protection from premature metabolism, prolonged systemic circulation and enhanced tumor accumulation via combined passive and active targeting. PCPs represent a versatile and underexploited toolbox for cancer drug delivery. Expanding the repertoire of protein carriers, applying in silico rational design to guide the selection and optimization of drug–protein complexes, and exploring the use of patient-derived autologous proteins may open new opportunities for more effective and personalized therapies.

## Simple Summary

Blood proteins naturally transport hydrophobic molecules such as fats, vitamins, and hormones throughout the body. This perspective explores how to hijack these natural delivery systems through the Trojan Horse Approach to enable the controlled transport of anticancer drugs, thereby enhancing their solubility, bioavailability, pharmacokinetic profile, and therapeutic efficacy. The potential advantages of this strategy include better control over drug circulation in the bloodstream, the prevention of early breakdown, and preferential tumor accumulation. In this perspective, we discuss how drugs are loaded onto these proteins and how this changes their journey through the body. The main aim is to explore the challenges and opportunities in the development of future therapies employing plasma carrier protein-based formulations.

## 1. Introduction

Chemotherapy remains one of the most widely used strategies for cancer treatment. However, many clinically approved chemotherapeutic drugs (CDs) are highly hydrophobic, display poor aqueous solubility, have short blood circulation half-lives, and undergo rapid systemic clearance. In several cases, their clinical administration requires toxic solubilizing excipients, such as ethanol, Cremophor^®^ EL, or Tween 80^®^. Moreover, CDs lack selective targeting, leading to widespread systemic distribution and consequent severe dose-limiting toxicities, including cardiomyopathy and myelosuppression. Consequently, the efficacy of chemotherapy is often compromised by the limited fraction of the administered drug reaching the tumor and by systemic adverse effects [1,2].

To overcome these issues, a wide range of nanosized delivery systems have been developed, including polymeric nanoparticles, liposomes, and inorganic nanocarriers [3,4]. Despite remarkable progress, these platforms still suffer from important drawbacks, notably (i) the variability in size, shape, and surface properties, (ii) the limited stability under physiological conditions, poor tumor specificity and cellular uptake, (iii) challenges in achieving uniform biodistribution, controlled drug release, biodegradation, and excretion, (iv) potential toxicity and immunogenicity and (v) the complex, expensive and time-consuming synthetic procedures [5,6].

In this context, proteins have emerged as exceptionally versatile supramolecular hosts owing to their intrinsic biocompatibility, biodegradability, low toxicity, prolonged circulation and well-defined molecular structures. In particular, PCPs are attractive scaffolds for the ex vivo preparation of supramolecular drug–protein complexes, where therapeutic agents are non-covalently associated with natural protein carriers prior to administration. Such formulations can improve the aqueous solubility and bioavailability of hydrophobic drugs, protect them from degradation and premature metabolism, prolong blood circulation, and promote tumor accumulation [7,8].

Currently, protein-based nanoparticles represent one of the most clinically advanced classes of protein nanomedicines. Several formulations have entered clinical trials, and two albumin-based nanoparticles have received FDA approval for systemic cancer therapy. The first, Abraxane^®^ (albumin-bound paclitaxel nanoparticles), approved in 2005 for breast, lung, and pancreatic cancers, exhibits lower systemic toxicity and improved clinical outcomes compared with solvent-based paclitaxel formulations [9,10,11,12]. More recently, Fyarro^®^ (albumin-bound sirolimus nanoparticles) was approved for the treatment of advanced malignant perivascular epithelioid cell tumors (PEComa), further validating protein-based nanoparticles as clinically successful drug delivery platforms. Nonetheless, protein nanoparticles still present several intrinsic limitations [13,14]. Their preparation generally yields heterogeneous and polydisperse assemblies, with limited control over protein–drug stoichiometry and drug loading. Moreover, manufacturing can alter the native conformation of the carrier, potentially affecting its biological properties [15,16,17].

It is critical to recognize that such non-covalently assembled protein nanoparticles undergo dynamic disassembly following intravenous administration, suggesting that individual protein molecules, rather than the nanoparticle itself, function as the actual delivery unit in vivo [11,18]. In this context, discrete drug–protein supramolecular complexes emerge as an attractive alternative to conventional protein nanoparticles. The potential advantages of such systems include a more precise control over composition, homogeneity and stoichiometry, enhanced reproducibility and a more predictable pharmacokinetic behavior. Furthermore, preserving the native structure and biological properties of the protein carrier prevents limitations due to the use of nanoparticles and ensures more reliable interactions with biological targets [19]. When the relevant biological properties of the endogenous carrier are preserved after drug loading, discrete drug–protein complexes may also be less readily recognized and cleared by the mononuclear phagocyte system/reticuloendothelial system (RES).

In this perspective, we examine the application of the Trojan Horse Approach (THA), based on endogenous plasma proteins, for cancer therapy. Particular emphasis is placed on the strategies for incorporating therapeutic agents into plasma protein carriers, the pharmacokinetic and pharmacodynamic principles governing the in vivo performance, and the mechanisms that enable selective tumor targeting. Finally, we provide a perspective on expanding the current repertoire of plasma protein carriers, guiding the rational design of next-generation supramolecular delivery systems, and advancing personalized precision oncology.

## 2. Plasma Carrier Proteins as Supramolecular Delivery Systems for Chemotherapeutics: The Trojan Horse Approach

The Trojan Horse Approach (THA) exploits proteins as discrete supramolecular hosts capable of reversibly accommodating therapeutic molecules while preserving the native structure and biological function of the carrier (Figure 1) [20,21]. Unlike conventional protein nanoparticles, each drug–protein complex ideally constitutes a single molecular entity with a well-defined composition and stoichiometry, predictable pharmacokinetic behavior, and straightforward physicochemical characterization. These features define the THA as a distinct paradigm for protein-based drug delivery, offering a level of molecular precision that cannot be achieved by conventional nanoparticle platforms. The molecular basis of THA lies in the exceptional host–guest recognition properties of proteins. Therapeutic agents can be accommodated within hydrophobic cavities, ligand-binding pockets, or surface recognition sites through the synergistic contribution of geometric and chemical complementarity [22,23,24]. The intrinsic three-dimensional preorganization of protein binding sites minimizes the entropic penalty associated with complex formation, allowing stable yet reversible drug binding while preserving the structural integrity of both the guest molecule and the protein host.

This supramolecular recognition enables poorly water-soluble drugs to remain in a monomeric, pharmacologically active state within a protected microenvironment, avoiding aggregation and premature degradation while preserving their therapeutic activity. Because THA does not require covalent modification of the carrier, it may potentially preserve the native conformation and biological function of the protein, allowing the retention of its physiological transport properties and interactions with endogenous receptors [25,26]. Ideally, a Trojan Horse should solubilize a poorly water-soluble drug and maintain it tightly associated with the carrier during circulation, potentially through kinetic trapping (i.e., a low k_off_ associated with the poor drug solubility in the physiological environment). The intact complex should then be preferentially internalized by tumor cells, where intracellular processing of the carrier protein promotes the release of the active drug at the site of action.

PCPs are particularly attractive because evolution has optimized them for the transport of structurally diverse hydrophobic endogenous and exogenous ligands through the bloodstream. Beyond this physiological role, decades of biochemical, structural, and pharmacological investigations have generated an extensive body of knowledge regarding their ligand-binding sites, receptor recognition mechanisms, conformational dynamics, and trafficking pathways [7,27].

### 2.1. Drug Loading

The successful development of THA not only relies on the structural properties of the carrier but also on the strategy used to associate the therapeutic payload with the protein. Unlike conventional nanomedicines, where drugs are often passively encapsulated within polymeric or lipid-based matrices, protein-based Trojan Horses are built upon specific molecular recognition with the plasma protein. The resulting drug–protein complexes can be prepared ex vivo under controlled conditions before administration, allowing precise control over complex formation while exploiting the intrinsic transport mechanisms of endogenous plasma proteins in vivo. Two complementary loading strategies can be used (Figure 2): molecular recognition and molecular anchoring.

#### 2.1.1. Molecular Recognition

Molecular recognition represents the most straightforward strategy for the preparation of supramolecular drug–protein complexes. Rather than relying on chemical conjugation or nanoparticle encapsulation, the therapeutic molecule can be directly accommodated within naturally occurring ligand-binding pockets. The formation of the complex is therefore entirely governed by reversible non-covalent interactions, including hydrophobic effects, hydrogen bonding, electrostatic interactions, π-π stacking, cation–π interactions, and van der Waals forces. The balance among these interactions determines both the affinity and the residence time of the drug within the protein, ultimately influencing its pharmacokinetic behavior and biological activity.

Complex formation, synthesis, and purification are straightforward, do not require chemical modification or the use of toxic solvents, and are cost-effective. Natural protein binding pockets, without modification, may host drugs through geometric and chemical complementarity. The release of the drug depends on the specific carrier–drug pair and may result from reversible dissociation in response to changes in local equilibrium, competition with endogenous ligands or biomolecules, and variations in the physicochemical environment. Following cellular uptake of the intact complex, drug release may also occur through intracellular dissociation and/or endosomal–lysosomal processing of the carrier protein.

An appropriate balance between binding, transport, and release is therefore essential. For a drug with appreciable aqueous solubility and only modest affinity or rapid dissociation kinetics, dilution after intravenous administration may indeed lead to rapid dissociation and redistribution, thereby reducing or eliminating the distinction between administering a preformed complex and administering the free drug in the presence of plasma proteins. The ideal Trojan Horse cargo is therefore a poorly water-soluble drug with a low propensity to dissociate from the carrier under physiological conditions, making ex vivo complex preparation a critical step. The optimal affinity and dissociation kinetics are consequently system-dependent and should be evaluated under physiologically relevant conditions, including in the presence of competing plasma components.

#### 2.1.2. Molecular Anchoring

Molecular anchoring exploits the high affinity and molecular recognition properties of selected ligands for a plasma protein to enable the delivery of therapeutic agents that otherwise display limited intrinsic affinity for that carrier. This strategy involves two conceptually distinct steps. First, the therapeutic cargo is covalently conjugated to an anchor ligand, typically through a linker designed to preserve or restore the pharmacological activity of the drug. Second, the resulting drug–anchor conjugate interacts non-covalently with a specific binding pocket of the carrier protein, yielding a supramolecular drug–anchor–protein complex.

Thus, although molecular anchoring requires covalent construction of the drug–anchor conjugate, it does not involve irreversible chemical modification of the plasma protein itself. The interaction between the anchor and the carrier remains non-covalent and can exploit the intrinsic ligand-binding properties of the endogenous protein. This feature distinguishes molecular anchoring from conventional covalent protein–drug conjugates, in which the therapeutic payload is permanently attached directly to the carrier.

Careful drug derivatization is essential to ensure that, following cellular uptake and release, the active parent drug is regenerated. This often requires a cleavable linker that remains stable in circulation but is cleaved after uptake by target cells, e.g., through intracellular enzymes, acidic endosomal/lysosomal conditions, or redox cues [28,29,30]. Non-cleavable linkers are viable when the intact conjugate is active or when cellular catabolism generates an active drug-containing metabolite [31].

Because the affinity of the interaction can be rationally tuned through linker length, hydrophobicity, and anchor chemistry, molecular anchoring provides a versatile platform for controlling plasma residence time, biodistribution, and drug release kinetics.

### 2.2. Pharmacokinetics and Pharmacodynamics

The therapeutic efficacy of anticancer agents not only depends on their intrinsic pharmacological activity but also on the pharmacokinetic processes. Pharmacokinetics and pharmacodynamics are central to understanding how anticancer drugs behave in the body and how they produce both therapeutic and toxic effects. Pharmacokinetics describes the absorption, distribution, metabolism, and excretion of a drug, whereas pharmacodynamics links drug exposure to biological response, including tumor cell kill and dose-limiting toxicity; in oncology, this relationship is especially important because most agents have a narrow therapeutic window and highly variable patient responses. Many CDs suffer from poor aqueous solubility, rapid systemic clearance, extensive metabolism, and dose-limiting toxicity, resulting in suboptimal therapeutic indices. Drug pharmacokinetics are affected by binding to proteins, thus making drug–protein interactions imperative to study. In fact, the interaction of many CDs with plasma proteins and the role of plasma proteins for the transport and biodistribution of drugs after administration have been extensively reviewed [32,33,34,35,36,37,38].

The ex vivo preparation of supramolecular drug–protein complexes provides an opportunity to modulate pharmacokinetic properties before administration by exploiting the natural transport functions of endogenous plasma proteins to regulate how these drugs are transported, taken up, and released, closely resembling their natural circulation in the bloodstream [39,40,41]. Consequently, the pharmacological behavior of the complex substantially differs from that of the free drug and reflects the combined properties of both the therapeutic molecule and its biomimetic carrier [42]. Non-covalent association of a hydrophobic cargo with a soluble plasma protein may increase its apparent solubility, namely the total drug concentration that can be maintained in a physiologically relevant aqueous medium as a soluble drug–protein complex. Ex vivo drug–protein complexes can improve the fate of anticancer agents by shielding them from metabolism, prolonging their systemic circulation and improving cellular uptake and targeting [43,44]. From a therapeutic perspective, this strategy may allow the use of lower overall drug doses while still achieving elevated concentrations at the intended site of action, preserving the active chemical form and avoiding off-target release, ultimately improving therapeutic efficacy with lower systemic toxicity compared to conventional chemotherapy methods. However, these potential benefits can be achieved only when carrier-mediated transport and uptake of the intact complex compensate for the reduced availability of freely diffusible drug and enable the release of an active drug species within the target tissue (Figure 3).

#### 2.2.1. Protection from Metabolism

One of the principal pharmacokinetic advantages of ex vivo-prepared supramolecular drug–protein complexes is their ability to protect therapeutic agents from premature metabolic degradation during systemic circulation. Within the protein, the drug becomes partially shielded from the surrounding aqueous environment and from direct interaction with metabolic enzymes. This can result in enhanced stability of labile compounds as well as prolonged persistence of the pharmacologically active species in vivo.

Most importantly, unlike conventional formulations in which drug–protein association occurs dynamically after injection, ex vivo assembly may ensure that a substantial fraction of the administered dose is already associated with the carrier at the time of injection, thereby providing immediate pharmacokinetic protection. Poor aqueous solubility of the cargo is a central prerequisite for this ideal scenario: without it, and in the absence of sufficiently slow dissociation kinetics, ex vivo loading may offer little advantage over administration of the free drug.

#### 2.2.2. Prolongation of Systemic Circulation

Extending blood circulation represents one of the fundamental advantages of plasma protein Trojan Horses. By combining reduced renal filtration, prolonged systemic residence, and physiological recycling mechanisms, ex vivo supramolecular drug–protein complexes maximize the opportunity for tumor targeting while minimizing premature elimination of the therapeutic payload. Many clinically relevant anticancer agents are rapidly eliminated from the bloodstream because of their relatively small molecular size, poor aqueous solubility, and extensive distribution into peripheral tissues. As a consequence, only a limited fraction of the administered dose reaches the tumor, often requiring repeated administration at high doses and increasing the risk of systemic toxicity [1,6,7,32,45,46,47,48].

The increase in the apparent molecular size of the drug–protein complex represents one of the principal mechanisms responsible for prolonged plasma residence. Small chemotherapeutic molecules are readily filtered through the glomerular membrane, whereas plasma proteins often exceed the renal filtration threshold. Consequently, formation of supramolecular complexes may substantially reduce renal clearance and prolong the systemic exposure of the associated therapeutic agent. Beyond their molecular size, plasma proteins also possess intrinsic biological mechanisms that further extend their circulation lifetime.

For ex vivo supramolecular drug–protein complexes, these physiological transport mechanisms may be retained by the therapeutic formulation. Since the drug is already associated with its carrier before administration, the pharmacokinetic advantages of the plasma protein can influence those of the payload, reducing the stochastic and often incomplete protein binding that occurs after injection of the free drug. As a result, prolonged circulation may increase the probability of tumor accumulation through passive and/or receptor-mediated targeting mechanisms, where these are operative, while also reducing fluctuations in plasma drug concentration, potentially improving exposure and therapeutic efficacy.

### 2.3. Cellular Uptake and Targeting

The THA can determine a profound impact on the tumor accumulation of the drug, cellular uptake, and intracellular trafficking. The unique advantage of PCPs lies in their ability to exploit endogenous biological pathways that have been refined through evolution, rather than relying solely on passive diffusion or nonspecific endocytic uptake of the free drug. Following systemic administration, supramolecular drug–protein complexes accumulate within tumor tissues through the combined action of passive and active targeting mechanisms (Figure 4).

#### 2.3.1. Passive Targeting

Passive targeting may represent the first stage in the biological accumulation of protein Trojan Horses within solid tumors [8]. Rapid and poorly organized angiogenesis produces blood vessels with enlarged endothelial fenestrations, discontinuous basement membranes, and impaired pericyte coverage, resulting in significantly increased vascular permeability compared with healthy tissues. At the same time, the absence of an efficient lymphatic drainage system within most solid tumors limits the removal of extravasated macromolecules, leading to their progressive accumulation in the tumor interstitium. These two phenomena collectively constitute the well-known enhanced permeability and retention (EPR) effect, which has long been considered one of the principal mechanisms responsible for the preferential accumulation of macromolecular therapeutics and nanomedicines in tumors [49].

Plasma protein-based Trojan Horses may benefit from passive tumor accumulation through several complementary features. First, the prolonged circulation of a stable drug–protein complex, relative to the free drug, may increase the duration of exposure to tumor vasculature. Second, the small dimensions of single-protein carriers, particularly compared with larger nanoparticles, may facilitate extravasation and interstitial diffusion. Third, provided that the drug remains associated with the carrier, the larger hydrodynamic dimensions of the complex relative to the free drug may reduce back-diffusion and favor retention within the tumor interstitium [50,51].

The structural architecture of the Trojan Horse is therefore an important determinant of passive targeting. However, an inherent trade-off exists: dimensions that facilitate diffusion through the tumor interstitium may also limit retention relative to larger macromolecular or nanoparticle systems. For instance, HSA (66 kDa; hydrodynamic diameter of approximately 7 nm) lies near the lower size range generally considered compatible with substantial EPR-mediated retention. Consequently, the contribution of passive accumulation is expected to vary among PCPs and to depend on the specific drug–carrier complex.

#### 2.3.2. Active Targeting

Active targeting exploits the endogenous biological functions of plasma proteins to achieve selective cellular uptake. Because rapidly proliferating cancer cells exhibit increased metabolic demands, they frequently overexpress receptors responsible for the uptake and recycling of circulating plasma proteins. By associating therapeutic agents with these physiological carriers, protein Trojan Horses can hijack naturally occurring transport pathways, thereby enhancing tumor selectivity. Importantly, receptor-mediated recognition not only increases cellular internalization but also determines the intracellular trafficking route, ultimately influencing drug release, subcellular localization, and therapeutic efficacy.

The diversity of receptor-mediated transport pathways available to different plasma proteins considerably expands the opportunities for rational carrier selection. Rather than functioning merely as passive solubilizing agents, plasma proteins actively dictate the biodistribution and cellular uptake of the associated therapeutic payload through highly evolved physiological recognition mechanisms. Consequently, the choice of the carrier protein should not be solely based on its drug-binding properties but also on the expression profile, intracellular trafficking, and recycling behavior of its cognate receptor in the target tissue. Understanding these receptor–protein interactions is therefore essential for the rational design of next-generation protein Trojan Horses with enhanced tumor specificity and optimized therapeutic performance.

## 3. The Trojan Horse Approach: State of the Art

The investigation of the interactions between drugs and plasma proteins is the most extensively developed area underlying the THA. However, only a limited subset of the synthesized complexes has been deliberately prepared ex vivo as a drug-delivery formulation. The synthesis, physicochemical characterization, and functional evaluation of discrete drug–protein complexes in relevant in vitro models represent a less investigated but still rapidly developing area of research. In turn, evidence from in vivo pharmacokinetic, biodistribution, and antitumor efficacy remains limited to selected systems, while clinical translation is still largely at a seminal stage. The following section distinguishes these non-interchangeable levels of evidence, which are presented in Table 1.

### 3.1. Molecular and Biophysical Demonstration of Drug Binding to PCPs

A wide range of molecular/biophysical studies provided evidence of drug–PCPs complex formation and their characterization. These studies have employed spectroscopic (fluorescence quenching, UV-Vis spectroscopy, circular dichroism, mass spectrometry), calorimetric, computational (docking, molecular dynamics) and structural techniques (NMR, X-ray crystallography). Only in a few cases have binding-site location of the anticancer agents been established by unequivocal structural evidence.

Human serum albumin (HSA) provides the best-characterized example among plasma proteins and its interaction with chemotherapeutic agents has been extensively investigated and reviewed elsewhere [32,33,34,35,36,37,52,53,54,55,56,57,58,59,60]. High-resolution X-ray crystallographic structures have directly resolved the interaction of clinically relevant compounds with HSA, including methotrexate, camptothecin, 9-aminocamptothecin, etoposide, teniposide, idarubicin, and cisplatin, thereby defining their binding modes and sites on the protein [37,59,60,61]. The most relevant examples of THA based on HSA were developed by Yu et al. who developed the so-called Single Protein Encapsulation (SPE) technology, used for the delivery of doxorubicin, SN38, epirubicin, daunorubicin, mitoxantrone, idarubicin, vincristine, vinblastine, and docetaxel [62,63,64,65].

Transferrin (Tf) was another extensively investigated protein, which formed supramolecular complexes with a variety of CDs, including artemisinin and its derivatives [66], temsirolimus [67], floxuridine [68], imatinib [69], leptosidin [70], and oxaliplatin [71], tamoxifen [72], hypocrellin A [73,74], doxorubicin and vanadocene [74].

Lactoferrin (Ltf) was reported to bind cisplatin [75] and kaempferol [76] whereas hemoglobin formed complexes with docetaxel [77], topotecan [78], amsacrine [79] and cisplatin [80].

The interactions of alpha-1-acid glycoprotein (AGP) with clofarabine [81] ponatinib, nintedanib [82], erlotinib [83], dacomitinib [84], sulfatinib [85], ibrutinib [86], apatinib [87] or gefitinib [88] were extensively characterized at the molecular level. However, in most cases the drug–protein assemblies were not subsequently isolated, purified, or developed as preloaded formulations. Only the paclitaxel–AGP complex was used as a Trojan Horse system for in vitro experiments [48].

Several drug–α-fetoprotein complexes have also been prepared [89,90]. Non-covalent interactions between Amphotericin B (AmB) and AFP resulted in the formation of the AmB–AFP complex [91]. Tetrachlorodibenzo-p-dioxin (TCDD) [92] and 1′-S-1′-acetoxychavicol acetate (ACA) [93] were also shown to bind AFP. Likewise, AIMPILA [94,95] was explicitly described as a non-covalent complex of porcine AFP and atractyloside, whereas patents reported ACT-901 and ACT-902 as reversibly bound complexes of AFP with paclitaxel [96] and thapsigargin [97], respectively.

In addition to conventional CDs, several blood proteins have been explored as carriers for photosensitizers [23,60,98,99]. For instance, hemopexin and haptoglobin can bind metalloporphyrins and phthalocyanines [100,101,102], whereas ceruloplasmin has been reported to carry chlorin e6 [103,104,105].

With regard to molecular anchoring, conjugation of CDs with albumin-binding motifs has emerged as an attractive strategy for exploiting endogenous albumin as a carrier. Representative examples include Evans Blue-modified derivatives [106] in which the albumin-binding moiety promotes reversible association with circulating albumin. Chemical derivatization with fatty acid moieties or natural albumin ligands has also been reported [107,108].

The most notable example of molecular anchoring strategy within the THA is a transthyretin (TTR)-based platform [30,109,110]. In this system, paclitaxel was conjugated through a spacer to a TTR-binding ligand derived from tafamidis, an FDA-approved drug for the treatment of ATTR amyloidosis. The resulting construct binds TTR within the thyroxine-binding pocket located at the interface between the A and B subunits of the TTR tetramer [109,111].

### 3.2. Synthesis and Physicochemical Characterization of Ex Vivo Prepared Drug–PCPs

Human serum albumin (HSA) was by far the most extensively characterized plasma carrier for Trojan Horse-based drug delivery [52,53,54,55,112,113,114,115,116,117,118,119]. Herein, the clearest examples of true ex vivo assembly were developed for doxorubicin [62,64,65,120,121] and SN-38 [63] delivery. In these studies, drug loading was achieved through multistep procedures in which parameters such as solvent composition, drug-to-protein ratio, and pH were controlled to favor incorporation of the hydrophobic payload into a single albumin molecule [62].

The complex formation was described using complementary analytical approaches, such as UV-Vis spectroscopy, fluorescence and KI-quenching experiments, and DLS, which indicated encapsulation of the drugs within monomeric HSA [62,63].

For SPEDOX complexes, dialysis experiments suggested a pH-responsive release mechanism that may become relevant following tumor-cell uptake and endosomal acidification. Compared with free doxorubicin and a simple DOX/HSA mixture, SPEDOX showed faster release under acidic conditions, consistent with weaker drug–protein binding at low pH [62,63]. A similar behavior was observed for SPESN38 relative to free SN-38 and a simple SN-38/HSA mixture. Notably, lyophilized SPESN38 complexes were reported to be highly water-soluble and to remain stable for more than 4 h at room temperature and for more than 24 h at 2–8 °C. They also retained stability for more than 4 h at room temperature in 4% HSA, a concentration comparable to that in human plasma [63].

For AFP-based systems [89,90,122], Pak et al. prepared supramolecular complexes by incubating AFP with amphotericin B and selected excipients, followed by sterile filtration and lyophilization, yielding a water-soluble formulation suitable for intravenous administration [91]. Likewise, AIMPILA was developed as an orally administered non-covalent atractyloside–AFP formulation [94,95]. Stronger physicochemical evidence was obtained for ACA-AFP. Ligand-free recombinant AFP was incubated with ACA at defined 1:1, 1:3, and 1:5 molar ratios, after which unbound ligand was removed by dialysis. Calorimetric analysis confirmed the formation of a distinct, reversible complex capable of solubilizing an otherwise poorly water-soluble molecule [93]. AFP-based formulations were also developed for paclitaxel and thapsigargin [96,97]. For paclitaxel, an optimized 4:1 drug-to-AFP formulation was reported to be soluble in water and saline, essentially free of uncomplexed drug, amenable to filtration, lyophilization, and reconstitution, and able to retain AFP secondary structure [96]. For thapsigargin, a 1:1 porcine AFP-to-drug complex was prepared by controlled incubation followed by ultrafiltration/diafiltration and freeze-drying, providing evidence of a preformed non-covalent delivery formulation [97].

Tf-based systems have also been explored as Trojan Horses. Artemisinin derivatives (ARTs) were incubated with Tf at defined molar ratios to generate ARTs–Tf adducts. The stability of the resulting assemblies was further examined in the presence of HSA, showing that a substantial fraction of artemisin–Tf remained associated under competitive conditions [66]. A particularly strong example is oxaliplatin–Tf, for which ex vivo formation of the intact complex was directly demonstrated using two orthogonal mass-spectrometric and chromatographic approaches [71].

Preformed cisplatin–Ltf formulations were prepared ex vivo in either 0.9% NaCl or 10 mM NaHCO_3_, pH 7.7. Characterization included FTIR, Raman, UV-Vis, fluorescence spectroscopy, DLS, electrophoresis, MALDI and SEM. The NaCl formulation predominantly retained a small-size protein population, whereas the NaHCO_3_ formulation showed marked aggregation [75]. By contrast, Ltf and kaempferol were simply mixed in PBS, and complex formation was assessed spectroscopically; no isolation, purification, or characterization of a defined complex was reported [76].

The apohemoglobin–haptoglobin (APH) platform represents a particularly advanced supramolecular formulation for the delivery of aluminum phthalocyanine. The payload was incorporated into the heme-binding sites of apohemoglobin and subsequently stabilized through association with haptoglobin to generate the final APH complex. The formulation showed controlled loading, a hydrodynamic diameter of approximately 18 nm, suppression of phthalocyanine aggregation, and retention of more than 80% of its characteristic absorbance after incubation in plasma for over 24 h at 37 °C. This level of characterization is important because it demonstrates not only molecular association but also preservation of a defined supramolecular construct under biologically relevant conditions, approaching the behavior expected of a protein-based nanocarrier [100].

The best-characterized molecular anchoring system is the tafamidis–paclitaxel conjugate bound to TTR. Its interaction with TTR was investigated by solution- and solid-state NMR spectroscopy and microscale thermophoresis, providing direct structural evidence of a stable, non-covalent drug–anchor–protein complex [109,111].

From a synthetic perspective, a particularly interesting approach was reported by Jaročytė et al., who synthesized Au nanoclusters directly in patient plasma, enabling the development of a personalized phototheranostic platform [123].

### 3.3. In Vitro Cellular Uptake and Functional Activity of Drug–PCPs

Transferrin represents the best example of receptor-mediated targeting for THA. Here, cellular uptake is primarily mediated by transferrin receptor 1 (TfR1), a membrane glycoprotein responsible for iron acquisition through clathrin-dependent endocytosis. Because iron is essential for DNA synthesis and cellular proliferation, TfR1 is markedly overexpressed in many malignant tumors, including breast, lung, colorectal, pancreatic, glioblastoma, and hematological cancers [51,124]. Following receptor binding, the transferrin-TfR1 complex undergoes rapid internalization into endosomes, where acidification promotes iron release while apo-transferrin remains associated with the receptor and is recycled back to the cell surface. This highly efficient uptake mechanism has made Tf a promising endogenous targeting ligand for enhanced intracellular drug delivery [124,125,126]. In HepG2 cells, Tf increased artesunate uptake by approximately 2- to 3-fold, depending on the Tf:Artemisin ratio, while leptosidin–Tf showed greater uptake in HT-29 cancer cells than free leptosidin and preferential accumulation over normal fibroblasts, consistent with TfR1-dependent selectivity [66,70]. Likewise, hypocrellin A–Tf was efficiently internalized by HeLa cells and subsequently localized in the mitochondria [73]. Overall, Tf complexes increased the activity of several payloads, including artemisinin derivatives, vanadocene, and leptosidin, supporting the potential contribution of increased intracellular delivery to enhanced pharmacological activity.

Tumor cells can actively take up HSA as a nutrient source to support their rapid growth [54,55,118]. Receptor-mediated drug delivery has also been investigated for albumin, for which gp60-mediated endothelial transcytosis and interactions with tumor-associated Secreted Protein Acidic and Rich in Cysteine (SPARC) have been proposed to contribute to transport and retention in selected tumors [53]. In the case of the SPEDOX system, immunofluorescence demonstrated a rapid cell internalization of the HSA-associated doxorubicin into BT-549 breast cancer cells, followed by nuclear accumulation of the drug. Uptake of albumin was markedly greater in proliferative cancer cells than in non-proliferative cardiomyocytes. This cell-type dependence provides a mechanistic explanation for both the preservation of antitumor activity and the reduced cardiotoxicity compared with free DOX [62,64,121].

Other endogenous plasma proteins may exploit additional uptake mechanisms, potentially broadening the Trojan Horse concept beyond the classical TfR1- and albumin-mediated pathways. Active delivery mechanisms were investigated for α-fetoprotein (AFP), whose receptor (AFPR) is highly expressed in many tumors but is largely absent in normal adult tissues. Tumor accumulation and therapeutic response of the AIMPILA complex were correlated with AFP receptor expression [94,95]. Similarly, the cytotoxic activity of ACA-AFP was correlated with AFP receptor expression [93]. However, although current evidence strongly supports AFPR-mediated targeting and receptor-associated delivery, direct quantitative evidence of intact-complex endocytosis remains limited for some formulations. Cellular uptake of cisplatin–Ltf complex was investigated in Caco-2 cells by immunocytochemistry and by intracellular platinum determination. After 3 h, the cisplatin-Ltf_in NaCl formulation produced significantly higher intracellular Pt accumulation than free cisplatin. The enhanced cellular uptake was associated with increased efficacy of the drug. MTT assay in Caco-2 cancer cells and L929 fibroblasts showed enhanced cytotoxicity of cisplatin–Ltf complex compared with cisplatin alone [75].

Kaempferol–Ltf cytotoxicity was evaluated in HeLa cells after 24 h of treatment. Free kaempferol exhibited an IC_50_ of 139.3 μM, whereas its combination with 3 μM Ltf reduced the IC_50_ to 101.6 μM. Annexin V–FITC/PI flow-cytometry analysis also showed increased apoptosis in cells treated with the complex compared with free kaempferol [76].

AGP-bound paclitaxel preserved the cytotoxicity of free paclitaxel [48], whereas AFP complexes such as ACA-AFP enhanced tumor-cell killing while sparing normal cells [93].

The APH platform further illustrated how the carrier architecture can modify cellular access to a payload, i.e., slowing down the Al-PC uptake compared to free drug [100]. Although this slower internalization is not intrinsically advantageous, it may support a more controlled route of cellular entry in selected systems. Protein carriers can also preserve stimulus-dependent activity, as shown by hypocrellin A-Tf and Al-PC-APH, which maintained low dark toxicity but strong phototoxicity upon irradiation [73,100]. Here, sequestration of the photosensitizer within the protein carrier reduced nonspecific membrane association and basal toxicity without compromising the photodynamic response.

### 3.4. In Vivo Pharmacokinetic and Biodistribution of Drug–PCPs

The binding of a drug inside a protein creates a favorable microenvironment preventing hydrolytic and oxidative degradation. This phenomenon was particularly relevant for camptothecin and its derivatives, whose active lactone form rapidly undergoes hydrolysis under physiological conditions. Structural and biophysical studies showed that the accommodation of camptothecin within the HSA binding sites stabilizes the lactone ring by limiting its exposure to water, thereby shifting the equilibrium toward the pharmacologically active species [127]. Similar protective effects were reported for several hydrophobic topoisomerase inhibitors and anthracyclines that associate with albumin through non-covalent interactions [62,63,120].

Similarly, mechanistic studies showed how Tf, APH, AGP and AFP can shelter labile [93] or hydrolysis-prone agents [90,94,122] from premature hydrolytic or oxidative pathways. This physical sequestration can help prevent photodegradation [73,88,100,103] and lower the immediately available free fraction of drug for hepatic uptake and cytochrome P450 metabolism, thereby prolonging systemic circulation.

The pharmacological consequence of protein complexation is not limited to a protection from metabolic inactivation but may also regulate clearance and enhance the control over the circulating free-drug fraction. Albumin represents the paradigmatic example, with an exceptionally long plasma half-life of approximately 19 days, resulting primarily from FcRn-mediated recycling, which continuously rescues internalized albumin from lysosomal degradation and returns it to the bloodstream. For HSA-based Trojan horse systems, protein complexation markedly improved drug disposition. SPEDOX increased circulating doxorubicin ~48-fold, with ~80% of blood DOX remaining protein-associated, while reducing free DOX in cardiac tissue by 4 to 8-fold, consistent with lower cardiotoxicity [62,64,121]. Similarly, SPESN38 altered the SN-38 pharmacokinetics and metabolism, increasing systemic exposure after intravenous administration and preserving the active parent drug by limiting glucuronidation [63].

Although HSA binding is well known to prolong drug circulation and systemic exposure [40,128], other plasma proteins may provide similar pharmacokinetic benefits. For example, artemisinin derivatives bound to transferrin showed prolonged circulation and preserved complex integrity during transport compared with the free drug [66]. This effect may be supported by TfR1-mediated recycling of transferrin, which limits immediate clearance while preserving its transport function. Following endosomal iron release, apo-transferrin remains associated with TfR1 and returns to the cell surface, where it is released into the circulation [28,29,30,111]. Likewise, AGP showed preferential tumor accumulation over HSA in C26-bearing mice, suggesting that the intrinsic biodistribution of the protein can influence the payload delivery [48]. More direct evidence was obtained with AIMPILA, which preferentially accumulated in AFP receptor-positive tumors, reaching a 4.5-fold higher level than normal mammary tissue at 24 h; its distribution closely paralleled that of AFP itself, supporting carrier-driven tumor targeting [94,95].

Reduced renal or extravascular loss has been proposed for the ~18 nm Al-PC–APH complex, and prolonged delivery has been suggested for platinum–Tf complexes, but direct in vivo pharmacokinetic confirmation remains limited [100].

### 3.5. In Vivo Antitumor Efficacy of Drug–PCPs

The ability of plasma-protein Trojan Horses to alter cellular selectivity was clearly reflected in vivo, where the therapeutic advantage is not only determined by a better tumor inhibition but also by the amount of drug that can be administered before dose-limiting systemic toxicity emerges.

In the SPEDOX complexes, albumin encapsulation enabled substantially higher DOX-equivalent dosing, with a cumulative 50 mg/kg dose producing no detectable cardiotoxicity in rats, consistent with reduced cardiac exposure [62,64]. Similarly, SPESN38 formulations achieved high tolerated doses and strong tumor regression with limited body-weight loss [63].

AFP-based ACA and paclitaxel formulations also improved the efficacy/toxicity balance. A low dose of paclitaxel–AFP outperformed even higher doses of free paclitaxel without overt toxicity, whereas high-dose paclitaxel was associated with a substantially less favorable toxicity profile [96]. These observations support the idea that AFP-mediated targeting can partially uncouple the dose required for tumor control from the dose producing systemic toxicity. Preclinical studies of the thapsigargin–AFP complex showed substantial tumor inhibition and prolonged survival in P-388 leukemia-bearing mice. In a colorectal cancer xenograft model, further experiments on this complex reported a complete tumor regression in most animals [97]. However, the TCDD–AFP example showed how protein binding did not automatically eliminate intrinsic payload toxicity. Here, while AFP complexation dramatically increased TCDD solubility and enhanced its biological activity, intravenous administration of the complex to mice did not abolish the intrinsic systemic toxicity of the dioxin. Indeed, toxicity remained comparable to that produced by free TCDD administered in an oil vehicle [92].

### 3.6. Clinical Evidence of Drug–PCPs

Currently, the most mature technology from the clinical point of view is the HSA-based SPEDOX formulation. Following extensive pharmacokinetic, efficacy, cardiotoxicity and GLP toxicology studies, SPEDOX received FDA Orphan Drug Designation for soft-tissue sarcoma and authorization under IND 152154 for a Phase Ib/IIa clinical study [65]. However, the available literature reports the study as ongoing and therefore does not yet provide human antitumor efficacy data. By comparison, the related SPESN38 formulations remained at the preclinical development stage, despite favorable solubility, pharmacokinetics, tolerability and xenograft efficacy, and have not yet generated clinical evidence [63].

Interestingly, some of the earliest AFP-based systems reached human administration despite having considerably less rigorous physicochemical characterization than modern HSA formulations. The non-covalent AFP–amphotericin B preparation was reported to have been administered to eight patients with stage IV malignancies progressing after conventional treatment. A full or partial clinical effect was reported in six of the eight patients, with remissions described as lasting approximately six months to 1.5 years. These observations are intriguing because they demonstrate that an injectable AFP-based non-covalent formulation reached human use [91].

Although only preliminary data are available, the AIMPILA complex also showed promising potential. In a small study of 12 patients with metastatic colorectal cancer and liver metastases, nine were evaluable according to RECIST after oral AIMPILA treatment. The study reported two patients with complete responses, one partial response, three cases of stable disease and three cases of progressive disease [129].

**Table 1:** A summary of the studies reporting the use of PCPs for the Trojan Horse Approach and corresponding levels of evidence investigated.

Protein	Drug	Drug Binding	Synthesis & Characterization	In Vitro Uptake & Activity	In Vivo PK & Biodistribution	In Vivo Antitumor Efficacy	Clinical Evidences	Patent	Ref
**Human Serum Albumin (HSA)**	**Doxorubicin**	✓	✓	✓	✓	✓	✓	✓	[62,64,65,121]
	**Daunorubicin**	✓	✓	X	X	X	X	✓	[62]
	**Mitoxantrone**	✓	✓	X	X	X	X	✓	[62]
	**Idarubicin**	✓	✓	X	X	X	X	✓	[62]
	**Vincristine**	✓	✓	X	X	X	X	✓	[62]
	**Vinblastine**	✓	✓	X	X	X	X	✓	[62]
	**SN-38**	✓	✓	✓	✓	✓	X	✓	[63]
**alpha-1-acid glycoprotein (AGP)**	**Paclitaxel**	X	✓	✓	X	X	X	X	[48]
	**Nintedanib**	✓	X	X	X	X	X	X	[82,83]
	**Clofarabine**	✓	X	X	X	X	X	X	[81]
	**Ponatinib**	✓	X	X	X	X	X	X	[83]
	**Erlotinib**	✓	X	X	X	X	X	X	[83]
	**KP2692**	✓	X	X	X	X	X	X	[83]
	**Dacomitinib**	✓	X	X	X	X	X	X	[84]
	**Sulfatinib**	✓	X	X	X	X	X	X	[85]
	**Ibrutinib**	✓	X	X	X	X	X	X	[86]
	**Apatinib**	✓	X	X	X	X	X	X	[87]
	**Gefitinib**	✓	X	X	X	X	X	X	[88]
**Ceruloplasmin**	**Chlorin e6**	✓	X	X	X	X	X	X	[103,104,105]
**Hemoglobin (Hb)**	**Amsacrine**	✓	X	X	X	X	X	X	[79]
	**Topotecan**		X	X	X	X	X	X	[78]
	**Docetaxel**	✓	X	X	X	X	X	X	[77]
	**Cisplatin**	✓	X	X	X	X	X	X	[80]
**alpha-** **fetoprotein (AFP)**	**AmphotericinB**	X	✓	✓	X	X	✓	✓	[91]
	**TCDD/dioxin**	✓	✓	✓	X	X	X	X	[92]
	**Atractyloside (AIMPILA)**	X	✓	✓	✓	✓	✓	✓	[94,95,129]
	**1′-S-1′-acetoxychavicol acetate (ACA)**	✓	✓	✓	X	✓	X	X	[93]
	**Paclitaxel (ACT-901)**	✓	✓	X	X	✓	✓	✓	[96,97]
	**Thapsigargin (ACT-902)**	X	✓	X	X	✓	X	✓	[97]
**Transferrin (Tf)**	**Artemisin (derivatives)**	✓	✓	✓	X	X	X	X	[66]
	**Vanadocene (Cp_2_VCl_2_)**	✓	✓	✓	X	X	X	X	[74]
	**Doxorubicin,**	✓	✓	✓	X	X	X	X	[74]
	**Tamoxifen**	✓	✓	X	X	X	X	X	[72]
	**Leptosidin**	✓	X	✓	X	X	X	X	[70]
	**Hypocrellin A**	✓	✓	✓	X	X	X	X	[73]
	**Oxaliplatin**	✓	✓	X	X	X	X	X	[71]
	**Temsirolimus**	✓	X	X	X	X	X	X	[67]
	**Floxuridine**	✓	X	X	X	X	X	X	[68]
	**Imatinib**	✓	X	X	X	X	X	X	[69]
**ApoHb-** **Haptoglobin (APH)**	**Aluminum phthalocyanine**	✓	✓	✓	X	X	X	X	[100]
**Transthyretin (TTR)**	**Tafamidis–paclitaxel**	✓	✓		X	X	X	X	[109,110]
**Lactoferrin (Ltf)**	**Cisplatin**	✓	✓	✓	X	X	X	X	[75]
	**Kaempferol**	✓	✓	✓	X	X	X	X	[76]

## 4. Future Perspectives: Advancing the Trojan Horse Concept for Precision Drug Delivery

Although the Trojan Horse Approach has already demonstrated considerable potential, its full impact will depend on the ability to broaden the repertoire of exploitable plasma proteins, rationally engineer next-generation carrier systems, and tailor these platforms to the biological characteristics of individual patients. The following sections discuss these emerging directions, which are expected to drive the evolution of plasma protein-based drug delivery toward precision medicine.

### 4.1. Expanding the Plasma Protein Toolbox

To date, the Trojan Horse Approach (THA) has exploited only a small fraction of the transport proteins naturally present in human plasma. This includes hundreds of proteins that have evolved to bind and transport lipids, hormones, vitamins, metal ions, metabolites, and numerous hydrophobic molecules with extraordinary affinity and selectivity. Rather than representing redundant transport systems, these proteins display substantial differences in three-dimensional architecture, ligand-binding mechanisms, receptor recognition, circulation half-life, tissue tropism, and intracellular trafficking. Such diversity provides an exceptional opportunity to expand the repertoire of endogenous carriers available for cancer therapy [19,45].

Broadening the repertoire of plasma-derived Trojan Horses could pave the way for a modular toolbox of endogenous carriers, enabling the rational selection of the optimal protein according to the physicochemical properties of the therapeutic payload, the biological characteristics of the target tissue, and the desired pharmacokinetic profile.

One promising direction is the exploration of proteins belonging to families that have already demonstrated successful carrier functions. Members of the albumin family, for example, possess conserved structural frameworks while displaying distinct ligand-binding preferences. Afamin, the physiological transporter of vitamin E, exemplifies this concept because, despite its close structural similarity to human serum albumin, afamin exhibits different binding characteristics. A similar concept applies to lactoferrin, which shares significant structural homology with transferrin while exhibiting distinct receptor interactions and biological activities, suggesting that homologous proteins may complement rather than simply replace existing carrier platforms. Ceruloplasmin, although much less investigated, possesses multiple metal-coordination sites that could be exploited for the transport of metal-based therapeutic agents [130,131].

Beyond structural homologues, another emerging strategy is the exploitation of highly specific endogenous ligand-binding pockets. Proteins such as retinol-binding protein (RBP) [132], corticosteroid-binding globulin (CBG), vitamin D-binding protein, and sex hormone-binding globulin (SHBG) [133] possess binding cavities that evolved to recognize their physiological ligands with exceptional affinity. Rather than relying on nonspecific hydrophobic interactions, these proteins could be repurposed through rationally designed molecular anchors that preserve high-affinity binding while carrying therapeutic payloads. Such an approach may reduce nonspecific drug partitioning into healthy tissues and improve pharmacokinetic control. Proof-of-concept studies already support this concept.

Besides drug loading, several plasma proteins possess intrinsic tissue-targeting capabilities: retinol-binding protein naturally targets hepatic stellate cells through the STRA6 receptor, a pathway that has already been exploited for selective delivery to fibrogenic liver cells. Importantly, this targeting mechanism can only be preserved when therapeutic molecules bind allosterically or through engineered molecular anchors without displacing retinol from its native binding site. Likewise, TTR has been reported to interact with receptors expressed by prostate and breast cancer cells, suggesting additional opportunities for receptor-mediated targeting. Hormone-binding globulins represent another attractive class of endogenous targeting proteins. Sex hormone-binding globulin (SHBG) interacts with membrane-associated SHBG receptors expressed in several hormone-sensitive malignancies, including prostate and breast cancer. Internalization of SHBG-ligand complexes through receptor-mediated endocytosis raises the intriguing possibility of exploiting this physiological pathway for selective intracellular drug delivery [133]. Although still at an early stage, these studies illustrate how endogenous endocrine transport pathways may be repurposed for precision oncology. Among alternative targeting proteins, lactoferrin is particularly attractive because it combines favorable drug-binding properties with broad receptor-mediated targeting capabilities. Lactoferrin interacts with receptors including LRP1, TLR4, CXCR4, and, in some contexts, intelectin-1, many of which are overexpressed in tumors or within the tumor microenvironment [134].

In addition to receptor-mediated uptake, some tumor cells can also internalize plasma proteins through other energy-dependent processes, such as micropinocytosis [135] and caveolae/lipid-raft-mediated endocytosis [136].

Macropinocytosis is particularly relevant in tumors with oncogenic Ras activation, in which it can promote plasma protein uptake and subsequent lysosomal degradation to sustain cellular nutrient requirements. In this context, plasma protein-associated therapeutic cargoes may potentially enter cancer cells through macropinocytic uptake [135,136].

The treatment of brain tumors represents a potentially valuable application for expanding the repertoire of plasma protein-based Trojan Horses. In general, plasma protein binding reduces the freely diffusible drug fraction and may therefore limit passive penetration across the blood–brain barrier (BBB). However, this limitation may be overcome by selected endogenous carriers that exploit receptor-mediated transport pathways. Transferrin is a well-established example, while lactoferrin represents a promising candidate that may also undergo receptor-mediated transcytosis across the BBB. Although the potential of transferrin- and lactoferrin-based non-covalent Trojan Horses remains largely unexplored, these proteins may provide opportunities for brain delivery, provided that the intact complex crosses the BBB efficiently and subsequently releases an active drug species within the central nervous system [137,138,139,140].

Rather than considering plasma proteins as isolated carrier candidates, future research should view the plasma proteome as a diverse library of naturally evolved transport platforms. Each protein combines unique ligand-binding characteristics, receptor interactions, tissue tropism, circulation kinetics, and intracellular trafficking pathways that have been optimized through evolution to solve specific biological transport problems. Harnessing this functional diversity would enable the rational selection of the most appropriate endogenous carrier for each therapeutic agent and clinical application. Such a transition from exploiting individual proteins to constructing a comprehensive plasma protein toolbox has the potential to transform the Trojan Horse Approach into a truly precision-oriented platform for cancer drug delivery, where carrier choice becomes as important as drug design itself (Figure 5).

### 4.2. Rational Design of Next-Generation Plasma Protein Trojan Horses

The expansion of the repertoire of exploitable plasma proteins represents only the first step toward the development of next-generation endogenous drug delivery systems. Equally important is the transition from empirical discovery to the rational design of supramolecular drug–protein complexes. Until now, most plasma protein carriers have been identified retrospectively, following observations that a given therapeutic agent binds a circulating protein with sufficient affinity to alter its pharmacokinetics or biodistribution. Recent advances in structural biology, artificial intelligence, and computational chemistry are rapidly transforming this paradigm, enabling the prospective design of drug–protein assemblies with predefined structural and functional properties.

The structural revolution driven by high-resolution cryo-electron microscopy and accelerated by deep learning-based prediction methods has substantially expanded the structural coverage of the human proteome. Experimental structures deposited in the Protein Data Bank continue to increase, while AlphaFold, RoseTTAFold, and related approaches have made structural models available for many proteins that had previously remained structurally uncharacterized. These resources can provide a valuable starting point for the investigation of plasma drug–protein interactions and for structure-guided carrier selection [98,141]. However, predicted structures should not be considered interchangeable with experimentally resolved, binding-competent conformations. Their utility depends on local model confidence and on biologically relevant features that may be incompletely represented, including protonation states, post-translational modifications, oligomeric state, endogenous ligand occupancy, and conformational heterogeneity.

Within these constraints, computational approaches may support the prioritization of plasma protein carriers for existing or newly synthesized therapeutic agents [98,141]. A realistic workflow would combine structural and physicochemical filtering with docking or virtual screening of candidate protein–drug pairs, followed by rescoring and the selective application of molecular dynamics simulations to assess binding-site accessibility, protein flexibility, and complex stability. More computationally demanding free-energy calculations should be reserved for a limited number of high-priority complexes rather than considered high-throughput screening tools. This hierarchical strategy may identify previously unrecognized supramolecular complexes and guide the selection of combinations for experimental testing. Conversely, starting from a selected plasma protein, virtual screening may help identify molecular anchors that favor stable drug–protein association while preserving, as far as possible, the physiological function of the endogenous carrier.

Beyond the identification of naturally occurring interactions, computational methods may eventually facilitate the rational engineering of new supramolecular systems. Advances in protein design, supported by machine learning and generative artificial intelligence, are increasingly enabling the redesign of ligand-binding pockets, the introduction of recognition motifs, and the modulation of receptor interactions. Such modifications could generate protein variants with improved affinity or selectivity for selected therapeutic cargos [142,143].

Another emerging opportunity is the integration of therapeutic and diagnostic functions within the same endogenous carrier. Several plasma proteins naturally bind metal ions, porphyrins, vitamins, or endogenous fluorophores, providing attractive platforms for the development of theranostic systems [144]. Albumin has already demonstrated the feasibility of simultaneously carrying imaging probes and therapeutic agents. The same supramolecular principles exploited for drug delivery could therefore be extended to the transport of MRI contrast agents, radionuclides, fluorescent probes, photosensitizers, or multimodal imaging agents, enabling simultaneous tumor visualization, image-guided therapy, and treatment monitoring.

Ultimately, the convergence of structural biology, computational protein design, artificial intelligence, and supramolecular chemistry is expected to transform the THA from a largely empirical strategy into a predictive engineering discipline. Rather than discovering plasma protein carriers by chance, future researchers may design them in silico, selecting or engineering the optimal endogenous transporter before experimental validation (Figure 6). This evolution has the potential to generate a new generation of plasma protein-based theranostic platforms capable of combining targeted drug delivery, molecular imaging, and precision medicine within a single supramolecular system.

### 4.3. Personalized Plasma Proteins for Precision Medicine

To date, plasma protein Trojan Horses have been developed primarily using commercially available or recombinant proteins. These approaches have established the feasibility of plasma protein-mediated drug delivery, while also suggesting a longer-term opportunity for precision nanomedicine: the potential use of circulating proteins isolated from individual patients as starting materials for personalized drug-carrier systems. In this hypothetical future scenario, selected plasma proteins could be isolated from patient plasma, associated ex vivo with a therapeutic cargo through supramolecular recognition or molecular anchoring, and subsequently administered to the same individual.

Such an approach could represent a conceptual shift from standardized nanocarriers toward patient-derived molecular delivery platforms. In principle, the use of plasma proteins derived from the intended recipient could reduce exposure to exogenous or recombinant carriers and preserve physiologically relevant protein isoforms and post-translational features. However, whether these potential advantages would persist after protein isolation, processing, drug loading, and reinfusion remains to be established. Likewise, naturally occurring glycosylation patterns and protein conformations might favor compatibility with endogenous transport pathways and receptor-mediated trafficking, but this possibility would require experimental validation for each drug–PCP complex.

Beyond possible immunological advantages, personalized plasma proteins could, in the longer term, enable carrier selection according to the biological characteristics of individual patients. The plasma proteome is not static but varies considerably among individuals owing to genetic background, age, sex, lifestyle, disease state, inflammation, and ongoing therapies. Protein abundance, post-translational modifications, ligand occupancy, and receptor expression can differ substantially between patients and may influence the pharmacokinetic and pharmacodynamic behavior of protein-based therapeutics. Although such variability currently represents a major translational challenge, future advances may allow selected patient-specific features to be considered as design parameters for individualized drug-delivery systems. Plasma proteomics, molecular docking, structural prediction, and artificial intelligence could eventually support the prioritization of candidate endogenous carrier–drug combinations for subsequent experimental validation (Figure 7).

The availability of multiple plasma proteins with distinct biological functions further supports the rationale for this hypothetical approach. Different carriers possess different circulation half-lives, tissue distributions, receptor interactions, intracellular trafficking pathways, and ligand-binding properties, and may therefore be investigated for distinct therapeutic objectives. Albumin could be considered for selected long-circulating hydrophobic drugs, whereas transferrin and other proteins could be evaluated when receptor-mediated uptake is of interest. Other plasma proteins may prove relevant for specific organs or molecular targets that remain unexplored. Thus, rather than relying on a universal carrier, future formulations might potentially select candidate plasma proteins according to both the physicochemical properties of the drug and the biological profile of the individual patient.

However, at present, this approach should be regarded as a conceptual future scenario rather than an actionable therapeutic strategy.

## 5. The Trojan Horse Approach: Current Limitations and Barriers to Translation

Despite their potential, plasma protein-based Trojan Horses face several interrelated limitations that may compromise their pharmacological performance and translational feasibility. These challenges not only concern the formation of the drug–protein complex, but also its stability in the biological environment, its ability to reach and enter the target tissue, the preservation of carrier function, and the reproducibility and scalability of its manufacture. Clinical translation remains at an early stage, and definitive evidence that this strategy improves clinical efficacy or the therapeutic index compared with conventional formulations will require well-controlled prospective clinical trials. Moreover, many relevant products and manufacturing procedures are patented, and their underlying data may remain unpublished or protected as confidential proprietary information.

### 5.1. Defining and Characterizing Drug–Protein Complexes

The available evidence for interactions between therapeutic agents and plasma proteins varies substantially, ranging from preliminary computational, spectroscopic, or equilibrium-binding studies to definitive structural characterization by X-ray crystallography or NMR spectroscopy. Importantly, the observation of binding in an equilibrium mixture does not necessarily demonstrate the formation of a sufficiently persistent, isolable, and administrable supramolecular formulation.

In favorable cases, particularly when the carrier contains a single dominant binding pocket or a limited number of well-characterized sites, defined stoichiometric drug–protein complexes can be prepared and experimentally verified. In other systems, however, the presence of multiple binding sites with different affinities may generate heterogeneous populations with distinct occupancy states. For example, HSA contains two major Sudlow sites in addition to several other hydrophobic binding regions. Therefore, drug association with HSA may result in a distribution of complexes rather than a single homogeneous species.

Under these conditions, drug loading should be described as an average drug-to-protein ratio rather than as a unique stoichiometry. Although this parameter can be modulated by the protein-to-drug ratio, concentration, incubation time, and purification procedure, the resulting distribution of species should be characterized under physiologically relevant conditions. Complex stoichiometry is further influenced by binding affinity and kinetics, competing endogenous ligands, protein heterogeneity, and the conditions used during ex vivo preparation.

### 5.2. Complex Stability, Cargo Release, and Preservation of Carrier Function

A main challenge is represented by achieving an appropriate balance between complex stability and cargo release. Premature dissociation may increase systemic exposure to the free drug and diminish the advantages of carrier-mediated delivery. Conversely, excessively stable interactions may hinder the release of an active therapeutic species at the intended site of action. This balance must therefore be assessed not only in simplified buffer systems but also in biologically relevant fluids and cellular environments.

Endogenous ligands and other plasma components may occupy carrier-binding sites, displace the therapeutic cargo, or promote protein exchange. In parallel, off-target interactions with circulating proteins, healthy tissues, or non-target cells may alter biodistribution and reduce selectivity. Protein degradation, proteolytic processing, renal or hepatic clearance, and exchange with endogenous proteins may further shorten systemic persistence or compromise complex integrity.

Drug binding or molecular anchoring may also perturb the biological properties of the carrier itself. Depending on the system, cargo loading can affect protein conformation, endogenous ligand binding, receptor recognition, oligomerization, intracellular trafficking, physiological function, and circulation properties. Consequently, predictable pharmacokinetic behavior cannot be solely inferred from the ex vivo formation of a defined complex. Rather, it should be demonstrated through dedicated studies addressing the stability, pharmacokinetics, biodistribution, metabolism, and therapeutic activity.

### 5.3. Limitations of Passive and Active Tumor Targeting

Neither passive nor active targeting can reliably guarantee the selective accumulation of a drug–protein complex within tumor tissues. The contribution of the EPR effect varies depending on tumor type, vascular architecture and permeability, stromal composition, interstitial pressure, disease stage, anatomical site, and experimental model. Quantitative studies indicate that only a small fraction of an administered nanomedicine dose typically reaches solid tumors, and that EPR-mediated accumulation often translates poorly from murine models to patients [145,146,147,148]. Passive accumulation should therefore be regarded as a possible contributor to the biodistribution of a given system rather than as a generalizable mechanism of tumor targeting.

Similarly, receptor-mediated uptake depends on several variables, including receptor abundance, accessibility, intratumoral heterogeneity, internalization kinetics, intracellular trafficking, and receptor expression in normal tissues. These features may differ among tumor types, across different regions of the same lesion, and during disease progression, thereby limiting the reliability and selectivity of receptor-targeted delivery strategies. Albumin-based delivery provides a prominent example of this complexity. These systems have been proposed to exploit endogenous pathways involved in albumin transport and cellular uptake. In selected experimental settings, interactions with endothelial gp60 have been associated with albumin transcytosis, whereas tumor-associated SPARC has been proposed to contribute to albumin retention or uptake in certain tumors [53,54,55,118].

However, these mechanisms should not be regarded as universal or independently sufficient determinants of tumor accumulation and therapeutic response. Their relative contribution is likely to depend on the specific albumin formulation, the physicochemical properties and release behavior of the associated cargo, and the biological features of the tumor microenvironment. Thus, receptor expression or albumin affinity alone does not demonstrate effective delivery of the intact therapeutic assembly to tumor cells.

Tumor cells may also internalize plasma protein-associated cargos through macropinocytosis, a receptor-independent pathway that is particularly active in some RAS-driven tumors. However, its therapeutic relevance is highly context-dependent, requiring a macropinocytic tumor phenotype, preservation of complex integrity during uptake, appropriate intracellular processing, and effective release of the active drug. In any case, many studies track the labeled carrier protein rather than the intact drug–protein assembly and should therefore be interpreted cautiously. Such experiments can provide strong evidence that the carrier enters tumor cells, but only indirect evidence that the complete complex remains intact during uptake and that the therapeutic cargo is delivered intracellularly. Whenever possible, both the carrier and the cargo should be monitored independently.

### 5.4. Translation from Experimental Models to Patients

Translation from experimental models to the clinical setting represents a further major hurdle. Tumor vascular permeability, stromal composition, interstitial pressure, perfusion, and receptor expression in animal models may not reproduce the heterogeneous and frequently poorly perfused microenvironment of human tumors. As a result, favorable accumulation, uptake, or efficacy observed in preclinical studies may not directly translate into clinical benefit [145,146,147,148].

Clinical evidence remains scarce, as only a limited number of plasma protein-based systems have entered clinical evaluation. Well-controlled prospective clinical trials will therefore be required to establish the actual translational value of the Trojan Horse Approach.

A further limitation is the lack of systematic head-to-head comparisons between discrete drug–protein Trojan Horses and established protein nanoparticle formulations. To date, no rigorous comparison of matched carrier–drug systems using standardized physicochemical, pharmacokinetic, biodistribution, tumor-accumulation, therapeutic-efficacy, and toxicity endpoints has been reported. Such studies will be essential to establish whether the reduced structural complexity of discrete drug–protein complexes provides tangible pharmacological or translational advantages over protein nanoparticles, including clinically approved formulations such as nab-paclitaxel.

In addition, the stability of the formulation during preparation, storage, transport, and administration must be rigorously established. A clinically viable Trojan Horse formulation requires reproducible drug loading, preservation of protein structure, controlled release behavior, and resistance to aggregation, degradation, and compositional changes over time.

### 5.5. Engineered and Personalized Plasma Protein Systems

Engineered plasma proteins cannot automatically be assumed to retain the favorable biocompatibility and pharmacokinetic properties of their native counterparts. Modifications affecting ligand-binding pockets, recognition motifs, or receptor interactions may alter protein folding and stability, immunogenicity, FcRn-mediated recycling, proteolytic susceptibility, biodistribution, and systemic clearance. The biochemical and pharmacological properties of each engineered variant must therefore be reassessed through appropriate structural, functional, and in vivo studies.

Personalized or autologous plasma protein formulations introduce additional translational complexity. A routine venipuncture provides only limited amounts of plasma and is unlikely to yield sufficient quantities of an individual carrier protein for many clinically relevant drug doses. Depending on the identity of the carrier, the intended drug-to-protein ratio, and the required dose, the preparation of a therapeutic formulation may require the collection of substantially larger plasma volumes by apheresis, followed by protein fractionation and purification. These requirements raise important questions concerning achievable carrier yield, collection frequency, processing time, cost, and the feasibility of repeated treatment cycles.

Additional complexity arises from the intrinsic variability of patient-derived proteins. Endogenous ligand occupancy and disease-associated alterations, including glycation, oxidation, proteolytic truncation, and inflammation-dependent modifications, may affect drug binding, complex stability, receptor interactions, and clearance. Protein isolation and drug loading may further modify the native structural or biological properties of the carrier, raising the question of whether the final drug–protein assembly should still be considered pharmacologically autologous.

Crucially, plasma-derived products require robust pathogen-safety procedures, including appropriate viral inactivation or pathogen-reduction strategies, as well as sterility assurance. These processes are generally associated with centralized, industrial-scale fractionation rather than simple bedside preparation and may themselves affect protein integrity and function. Moreover, an autologous carrier loaded with a cytotoxic drug constitutes a patient-specific drug product, requiring GMP-compatible manufacturing, analytical characterization, predefined release criteria, and individual batch release before administration. Quality control must therefore encompass not only carrier identity, purity, and stability, but also drug loading, residual free drug, complex integrity, sterility, endotoxin levels, and pathogen safety.

Systematic biochemical, pharmacological, manufacturing, and regulatory studies will consequently be required to establish whether personalized plasma protein Trojan Horses can be produced at clinically meaningful scale, with acceptable reproducibility, safety, and cost.

Overall, the development of clinically viable plasma protein Trojan Horses will require rigorous demonstration of complex identity, stability, release, safety, biodistribution, and therapeutic benefit, together with robust and reproducible manufacturing processes, stringent quality-control criteria, and clearly defined regulatory pathways.

## 6. Conclusions

The Trojan Horse Approach represents a distinct paradigm in protein-based drug delivery, in which endogenous PCPs are exploited as discrete supramolecular nanovectors rather than assembled into conventional nanoparticles. By offering the possibility of preserving the native structure and biological functions of the carrier, these systems combine the molecular precision of well-defined drug–protein complexes with the potential pharmacokinetic and targeting advantages that plasma proteins have evolved for the transport of endogenous ligands. As discussed throughout this perspective, ex vivo assembly through molecular recognition or molecular anchoring can improve drug solubility, protect therapeutic agents from premature metabolism, and prolong systemic circulation. Depending on the specific drug–protein system and its biological context, these properties may also favor tumor accumulation through passive and/or receptor-mediated mechanisms. Looking ahead, the greatest opportunity lies in moving beyond the current reliance on a few well-established proteins toward a broader and more rational exploitation of the plasma proteome. The increasing availability of structural information, together with advances in computational modeling, artificial intelligence, and protein engineering, is expected to transform the discovery of plasma protein carriers from an empirical process into a predictive design strategy. In parallel, the possibility of generating autologous drug–protein formulations from patient-derived plasma proteins opens an exciting route toward truly personalized drug delivery. Together, these developments may establish plasma protein Trojan Horses as a versatile platform for next-generation precision oncology, in which both the carrier and the therapeutic molecule are carefully selected and designed. Realizing this potential will require careful management of key limitations, including complex stability and drug release, competition with endogenous ligands, off-target interactions, biological variability, formulation robustness, and the manufacturing and regulatory challenges associated with clinically translatable drug–protein systems.

## Figures and Tables

**Figure 1:**
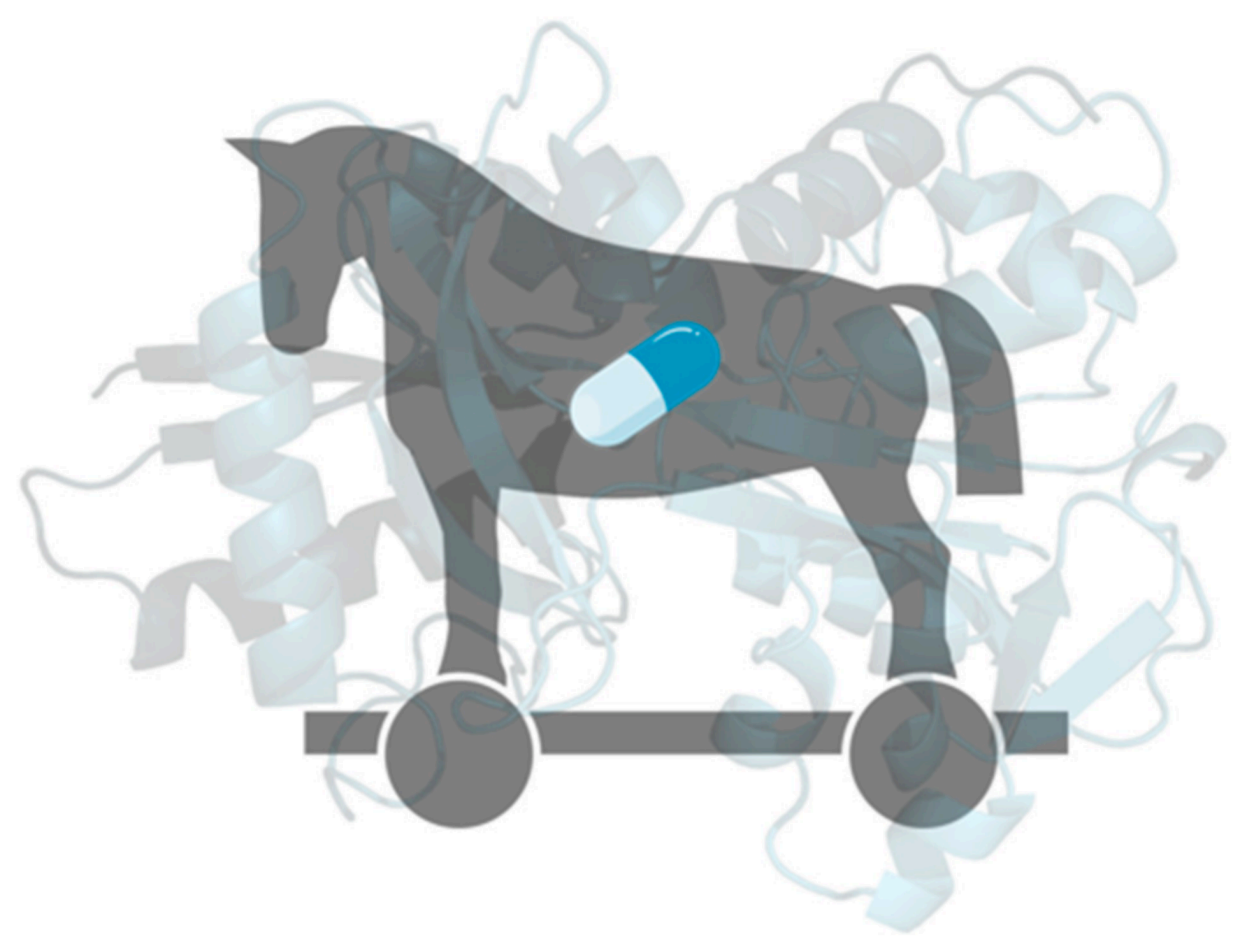
Illustration of the protein-based “Trojan Horse” approach for anticancer drug vehiculation.

**Figure 2:**
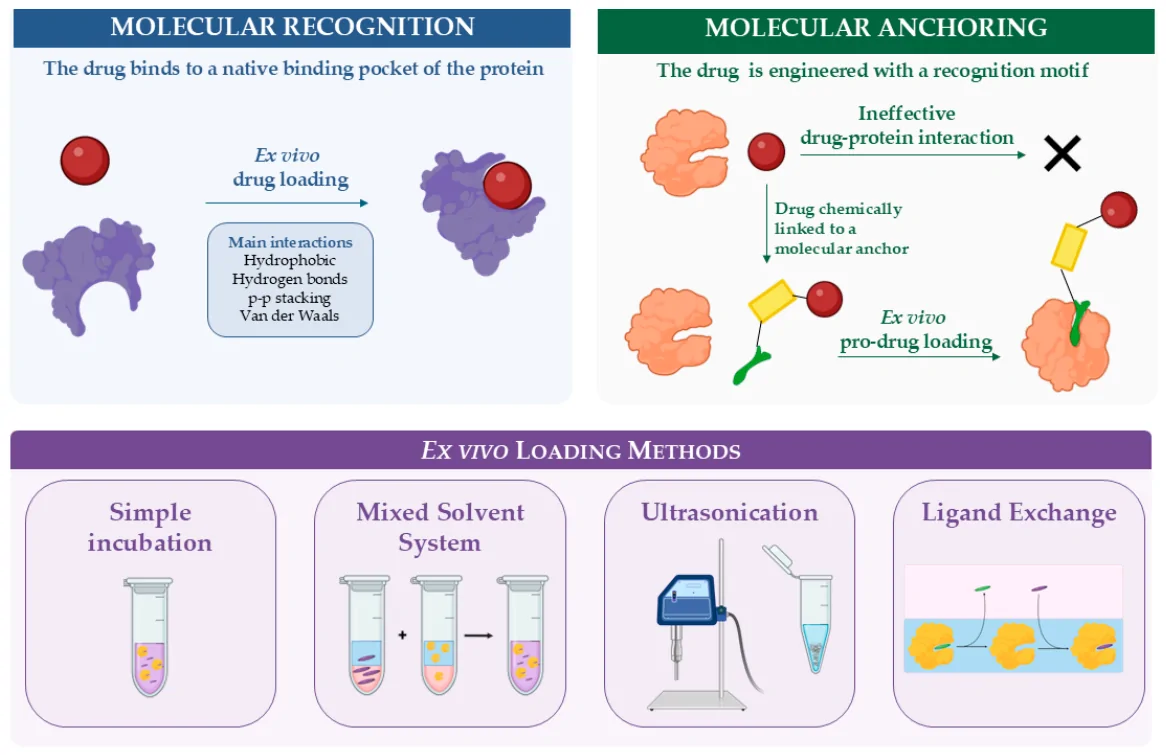
Schematic representation of the two main drug–protein interaction strategies (**top panel**) and loading methods (**bottom panel**) used to generate supramolecular drug–protein complexes within the Trojan Horse Approach (THA). Molecular recognition (**left**) exploits the intrinsic ligand-binding pockets of PCPs to directly and reversibly accommodate a therapeutic agent. Molecular anchoring (**right**) relies on chemical conjugation of the drug to a high-affinity anchor ligand that is non-covalently recognized by the binding site of the protein, enabling delivery of drugs that otherwise show poor intrinsic affinity for the carrier.

**Figure 3:**
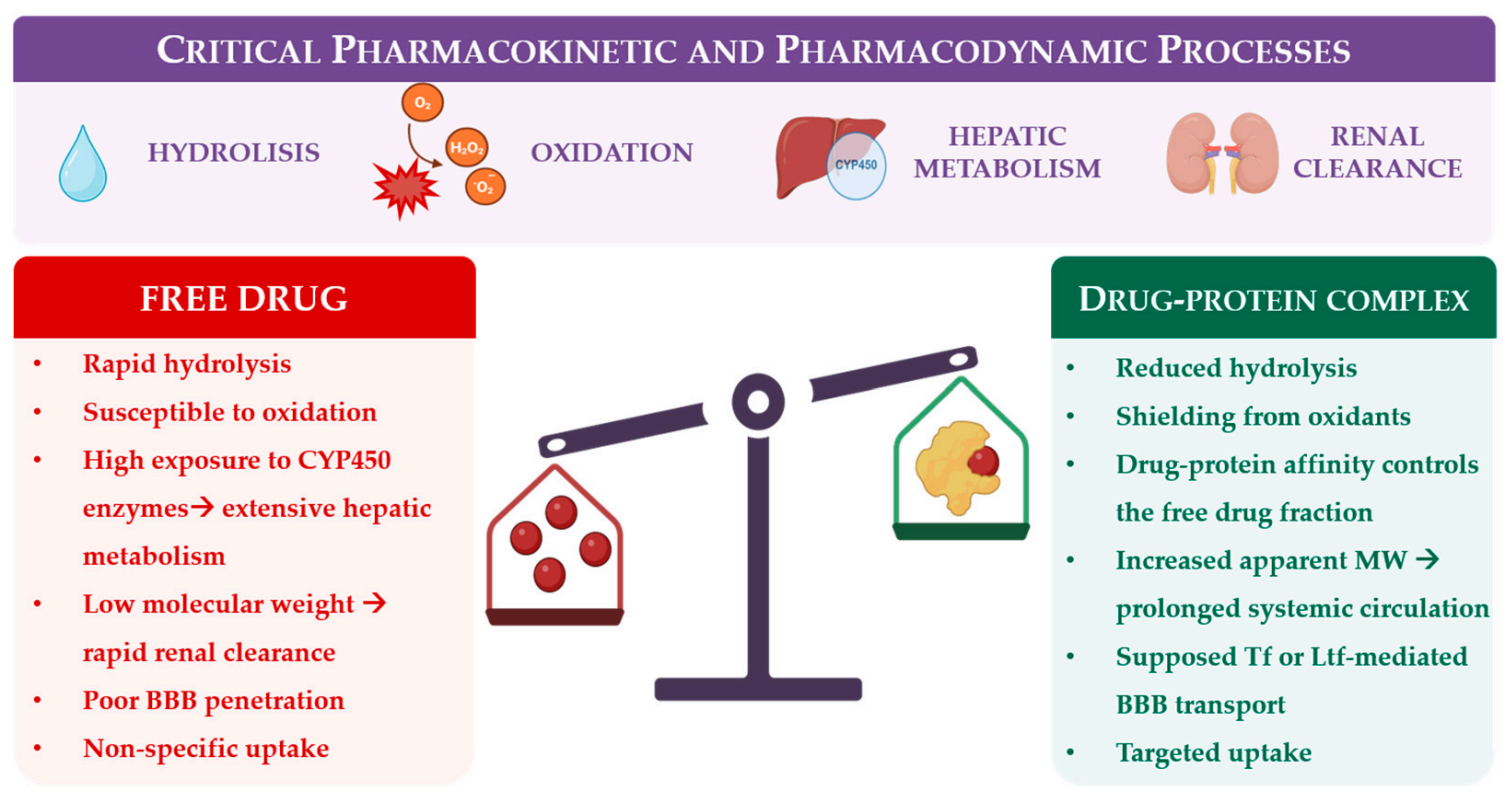
Mechanistic overview of the pharmacokinetic benefits potentially conferred by the Trojan Horse Approach. Binding of anticancer drugs to endogenous plasma proteins protects the therapeutic cargo from hydrolysis, oxidation, hepatic metabolism, and rapid renal clearance, thereby governing systemic exposure and circulation time.

**Figure 4:**
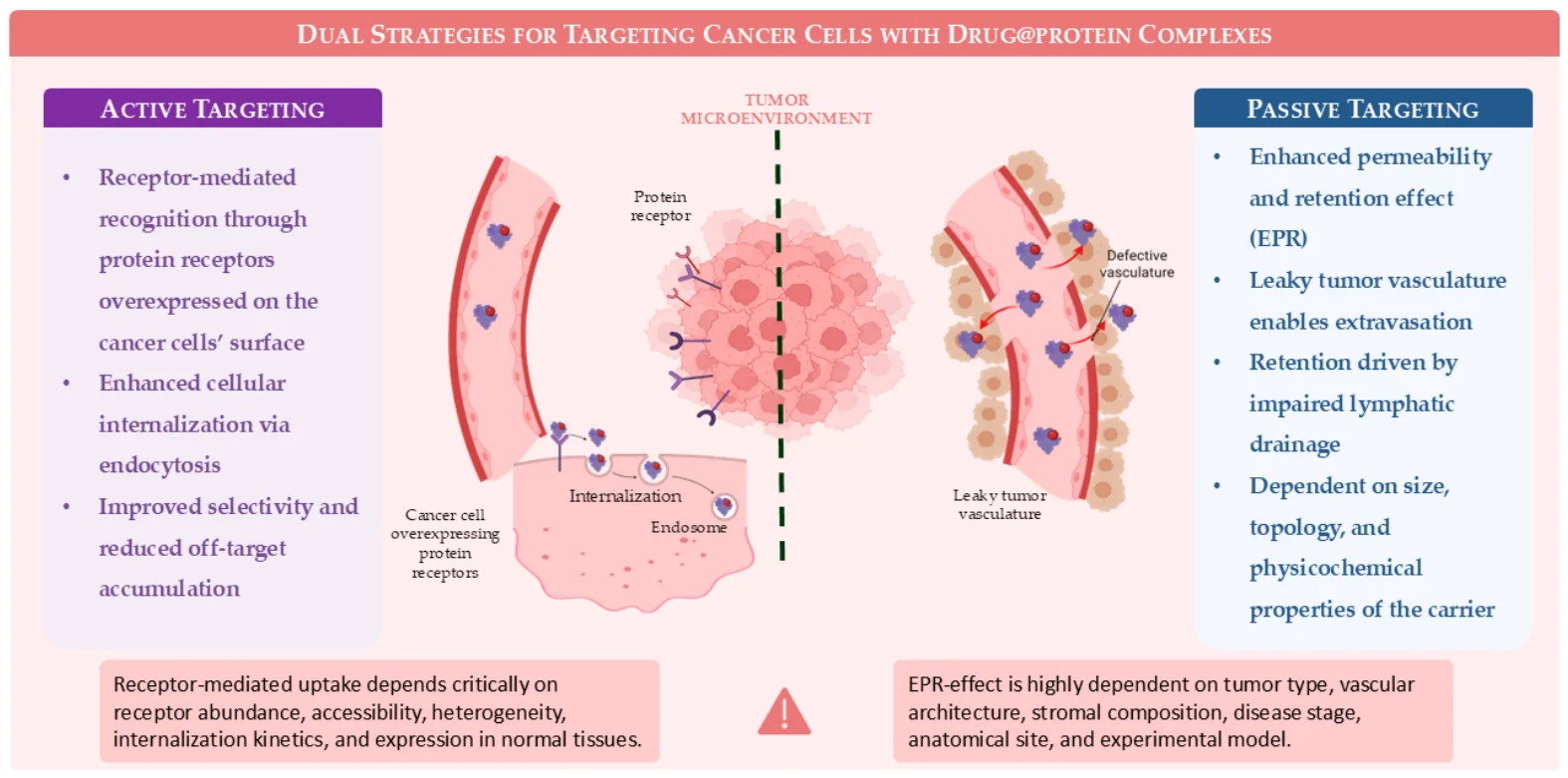
Context-dependent mechanisms potentially contributing to tumor accumulation exploited by plasma protein Trojan Horses. Passive targeting arises from the enhanced permeability and retention (EPR) effect associated with leaky tumor vasculature and impaired lymphatic drainage, while active targeting is mediated by receptors overexpressed on cancer cells that recognize specific plasma proteins, jointly improving cellular uptake and tumor selectivity.

**Figure 5:**
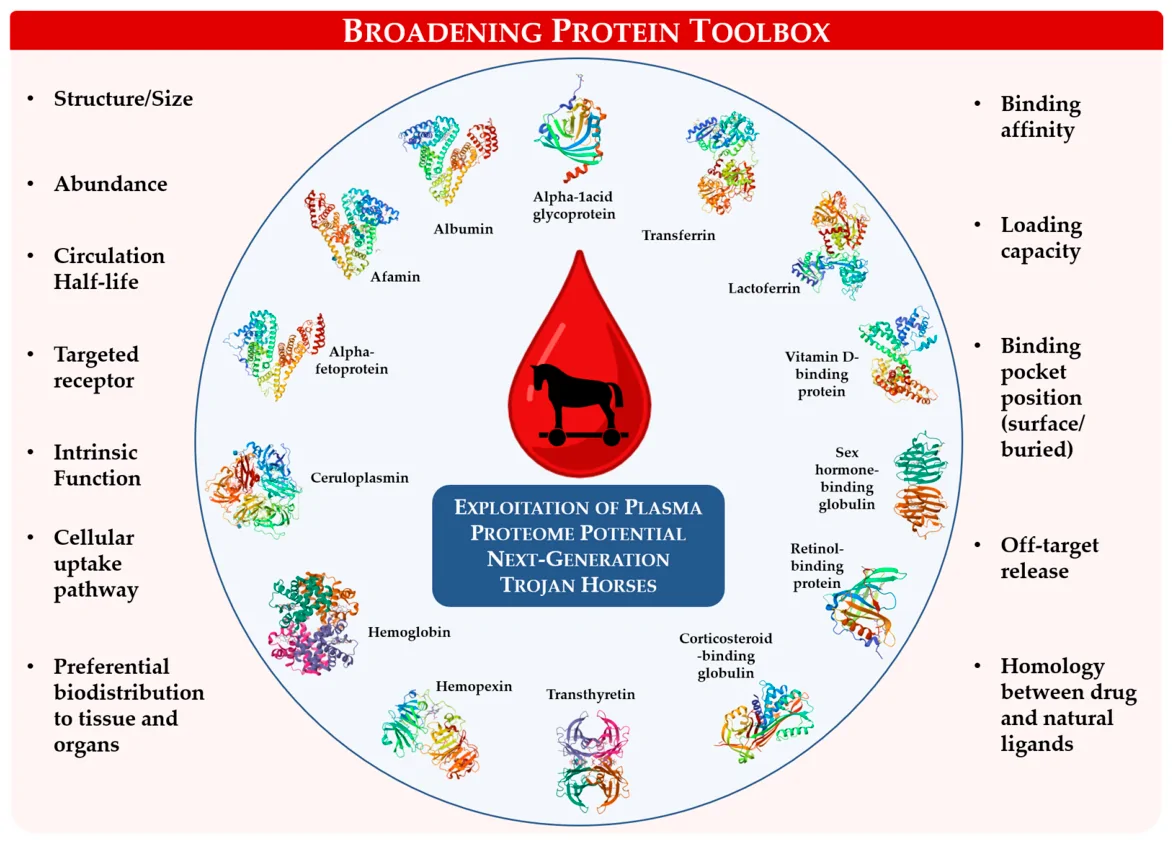
Key criteria guiding the rational selection of plasma carrier proteins for the design of next-generation Trojan Horses, including protein structure, ligand-binding affinity, plasma abundance, receptor recognition, and physiological function. The scheme highlights how these features must be evaluated jointly to identify the most suitable carrier beyond the classical albumin and transferrin platforms, also accounting for tissue tropism and the distinct intracellular trafficking pathways of each protein.

**Figure 6:**
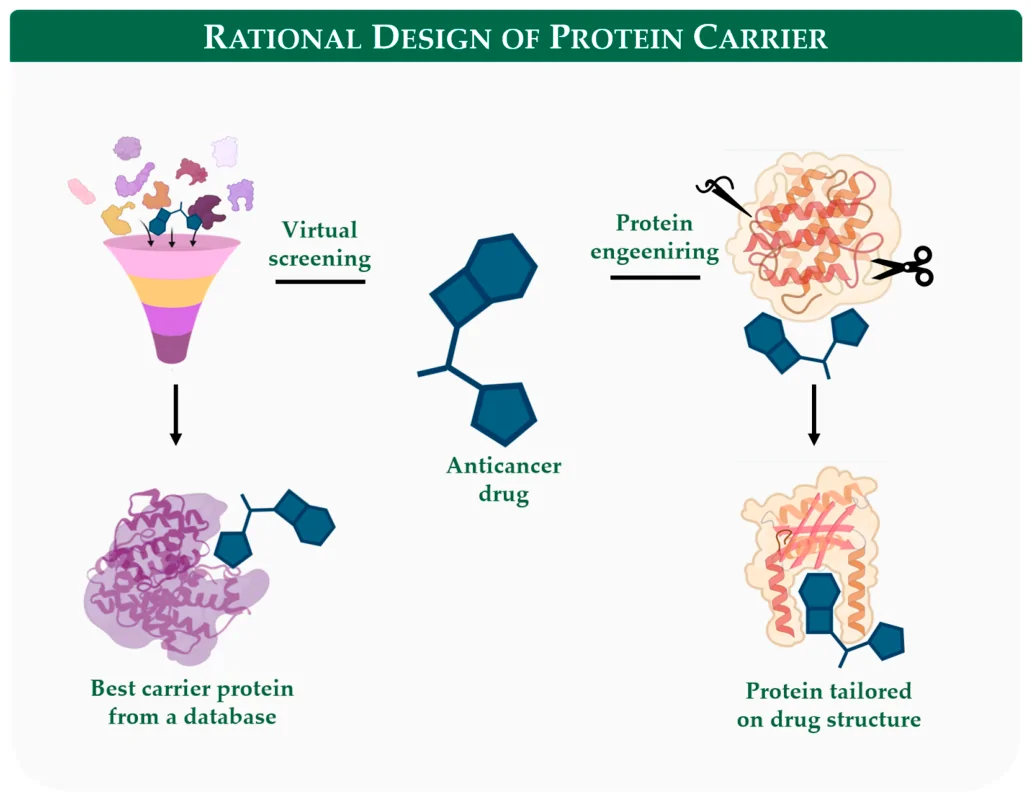
Conceptual workflow for the structure-guided and computationally assisted rational design of next-generation drug–protein complexes. The approach integrates high-resolution structural data, virtual screening methodologies (**left**) and AI-based predictive models (**right**) to prospectively identify or engineer the optimal plasma protein carrier for a given drug.

**Figure 7:**
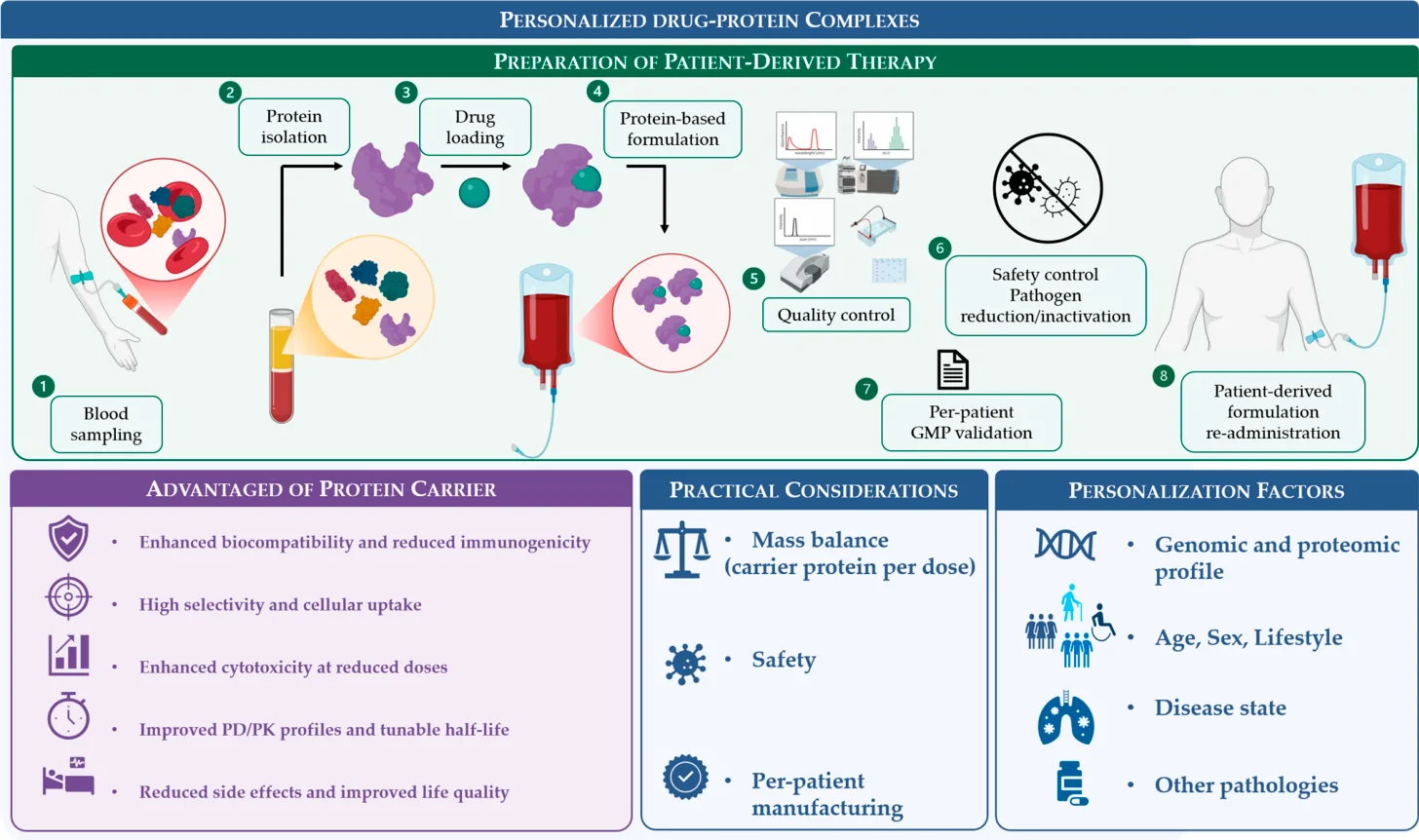
Conceptual future workflow for the hypothetical development of patient-derived plasma protein-based drug carriers. Carrier proteins could be isolated from the plasma of the patient, loaded ex vivo with a therapeutic agent through molecular recognition or molecular anchoring, and reinfused into the same patient as an autologous drug–protein complex. This approach may help to minimize immunogenicity, preserve the native post-translational modifications of the carrier, and improve biocompatibility, selectivity, and overall therapeutic outcome, thereby potentially paving the way toward truly personalized nanomedicine.

## Data Availability

No new data were created or analyzed in this study. Data sharing is not applicable to this article.
